# Design and validation of a rapid visual processing measure for screening reading difficulties in early childhood

**DOI:** 10.3758/s13428-025-02739-7

**Published:** 2025-07-25

**Authors:** Mahalakshmi Ramamurthy, Klint Kanopka, Adam Richie-Halford, Benjamin W. Domingue, Francesca Pei, Phaedra Bell, Lucy Yan, Andrea Hartsough, Maria Luisa Gorno-Tempini, Jason D. Yeatman

**Affiliations:** 1 Developmental-Behavioral Pediatrics, School of Medicine & Graduate School of Education, Stanford University, Stanford, CA USA; 2 Weill Institute for Neurosciences, University of California, San Francisco, CA USA

**Keywords:** Reading disabilities, Early visual processing abilities, Measure development, Item response theory, Early childhood

## Abstract

As the development of visual processing abilities is known to precede the development of reading abilities, identifying visual measures that reliably correlate with reading measures has tremendous theoretical and practical importance. A major challenge in addressing this question empirically is developing reliable behavioral measures for developmental studies, because most tasks are not equally reliable across different age groups and require iterative design changes to ensure that the measure reliably indexes the intended construct across the developmental span. Here, we present a series of studies that show how to iteratively modify a behavioral task by data-informed task changes and the use of item response theory to reduce task redundancies and develop a fun, fast, reliable, and easy-to-deploy web-based measure for K/1/2-graders in school settings. Our results show, in a large, diverse, and representative sample (*N* ~ 1,550), that the ability to rapidly encode visual information reliably correlates with reading outcomes at the end of the academic year. The developed measure offers the potential for use as an early screening tool to identify children at risk for developing future reading challenges.

**Supplementary Information:**

The online version contains supplementary material available at 10.3758/s13428-025-02739-7.

## Introduction

Task reliability is a fundamental aspect of behavioral and clinical research and is often a major challenge in the study of child development. It is particularly challenging to develop behavioral measures for developmental studies because most tasks are not equally reliable across different age groups and require iterative design changes to ensure that the measure reliably indexes the intended construct across the developmental span. Oftentimes a well-designed task that yields reliable data in adults might not in children. A classic example is in the design of vision charts to assess visual acuity. Visual acuity assessment is foundational to understanding the progress of several diseases and disorders that manifest as poor visual performance and therefore poor quality of life. Decades worth of research has converged on vision charts like the Snellen, Bailey Lovie, and Early Treatment of Diabetic Retinopathy Study (ETDRS) charts as gold-standard measures of visual acuity (Currie et al., 2000; Ferris et al., 1982). However, the same assessment loses reliability when extended to very young children (McGraw et al., 2000; Teller et al., 1986), thereby creating the need to create developmentally appropriate alternative measures. Preferential looking tests for infants (Mayer & Dobson, 1982), letter-matching tasks (Hedin et al., 1980), and single-letter acuity measures such as the Sheridan Gardiner test and Sight Test for Young Children and Retardates (STYCAR) were specifically designed and validated in young children and remain the primary measures of visual acuity in preschool children (Sheridan, n.d.; Stewart-Brown et al., 1988). Without these alternate measures it would not be possible to understand the development of the visual system and to detect visual anomalies early in development, without which early interventions would not be possible. Reliable tasks are therefore not only necessary for testing and refining scientific theory but can also serve as good screening and diagnostic tools.

In studying reading development and developmental dyslexia (DD)—a common reading disability with a global prevalence of 5–20% (Wagner et al., 2020)—a long-standing unresolved question is in understanding the relationship between individual differences in visual processing and reading development. A wealth of data have established correlations between various measures of visual processing and reading development (Facoetti et al., 2005, 2006; Franceschini et al., 2012; Gori & Facoetti, 2015; Joo et al., 2018; Rey et al., 2002; Saksida et al., 2016; Stein & Walsh, 1997; Talcott et al., 2000; White et al., 2019). However, the field has yet to reach consensus because existing theory on sensory processing in DD is built upon small samples (O’Brien & Yeatman, 2021; Talcott et al., 2002), using experimental measures without established psychometric properties, which are deployed across a wide age range, often without prior work demonstrating the validity of the measure in each developmental window. Many paradigms that work for adults (or older children) do not translate well to younger children. Moreover, the generalizability of any finding depends on the representativeness of the sample, and most vision science research depends on samples of convenience recruited from the surrounding university community. Without establishing the reliability and validity of behavioral measures in younger children, it is hard to derive meaningful theory linking various sensory processing mechanisms and reading development. Moreover, without designing and validating measures in an ecologically valid school setting, it is impossible to evaluate their utility for early screening.

Resolving the role of visual processing in the dyslexia phenotype is of great theoretical and practical importance. From the perspective of theory, the field is transitioning from “core deficit” models of dyslexia (Snowling, 1998; Vellutino et al., 2004) to multifactorial models that consider the combined influence of myriad risk factors and protective factors (Catts & Petscher, 2022; O’Brien & Yeatman, 2021). There is mounting evidence implicating various aspects of rapid and dynamic visual processing in the context of multifactorial models (O’Brien & Yeatman, 2021; Valdois, 2022), but there are also many unresolved details such as the factor structure of visual processing, phonological processing, language, and executive functions. From the perspective of practice, over 40 US states have passed dyslexia screening legislation requiring universal screening of kindergarten, first grade, and second grade students for word reading difficulties and dyslexia risk factors. A major challenge of early screening is distinguishing language experience (e.g., in multilingual learners) and early learning opportunities (e.g., opportunities to learn letters in preschool) from true biological risks for dyslexia. Validated measures of visual processing that predict future reading development could contribute to overcoming the challenges faced by other screening measures, since visual development is language-agnostic and not directly taught in preschool.

In this study, we leverage technology to scale precise laboratory measures into the classroom environment, using web applications (Bridges et al., 2020; de Leeuw et al., 2023). We develop and validate online measures of rapid visual processing in young children to be used in a school assessment setting. Specifically, we focus on translating a measure of rapid visual processing that has been shown to be relevant for reading in older children and adults in laboratory studies into a reliable measure for children as young as kindergartens in California public schools. We accomplish this goal through a series of studies that systematically investigate how the classic multi-element processing task (Ramamurthy et al., 2021; Sperling, 1960; Valdois, 2022) can be introduced through an online game to children in school settings, while maintaining the rigor of the laboratory testing. Our broader goal is to understand the association between individual differences in visual processing and reading development. If early visual processing abilities can reliably predict future reading difficulties, we could arrive at a potential language-agnostic early screener for future reading challenges. But the first step towards this larger goal is to develop and validate a reliable measure for young children.

### Online measure of rapid visual processing relevant for reading: Multi-element processing (MEP) task

In the partial report version of the multi-element processing (MEP) task, a string of letters, symbols, or any other visual element is briefly flashed at the center of the screen, and then the participant is cued to report the identity of a single randomly chosen element. This task taps into the amount of visual information that can be encoded in a brief glimpse (Sperling, 1960, 1983). Of all the measures of sensory processing that have been studied in relation to word reading difficulties, the multi-element processing task has the strongest evidence for identifying a subgroup of struggling readers who are not captured by conventional measures of phonological awareness. Previous studies have demonstrated that this task (a) correlates with reading ability (Bosse & Valdois, 2009; Ramamurthy et al., 2023), (b) differs in children with dyslexia (Lobier et al., 2012; Ramamurthy et al., 2023), (c) cannot be explained as a consequence of dyslexia (Lobier & Valdois, 2015), (d) might be a useful intervention target (Valdois et al., 2014; Zhao et al., 2019; Zoubrinetzky et al., 2019), and (e) identifies a subset of poor readers that have high phonological awareness despite their reading difficulties (Saksida et al., 2016; Valdois et al., 2020). Recent evidence suggests that this effect is consistent across languages including French, English, Dutch, and Chinese (Huang et al., 2021; Lobier & Valdois, 2015).

In a previous study (Ramamurthy et al., 2023), a 12-alternative forced-choice (12 AFC) multi-letter processing task was administered to a cross-sectional sample of 187 children aged 6–17 years. Across this age range, average task performance was 37.148% ± 1.191 correct, with reported reliability of 0.805. Moreover, performance in the multi-letter processing task correlated with reading ability across the whole age range (*n* = 187, *r* = 0.36, *p* = 3.9 × 10^−7^). This task, therefore, offers promise for investigating how the ability to rapidly encode visual information and retrieve relative positions of elements within a string relates to one’s reading abilities. However, to understand whether this task has the potential for an early dyslexia screener, it is critical to first be able to extend the same task to younger children (kindergarten and first grade, K/1) and establish good reliability so that longitudinal investigations are meaningful. We started out by first asking whether the current version of the task administered in the cross-sectional sample could be extended to a younger population with similar accuracy and reliability.

## General methods

**Fig. 1:**
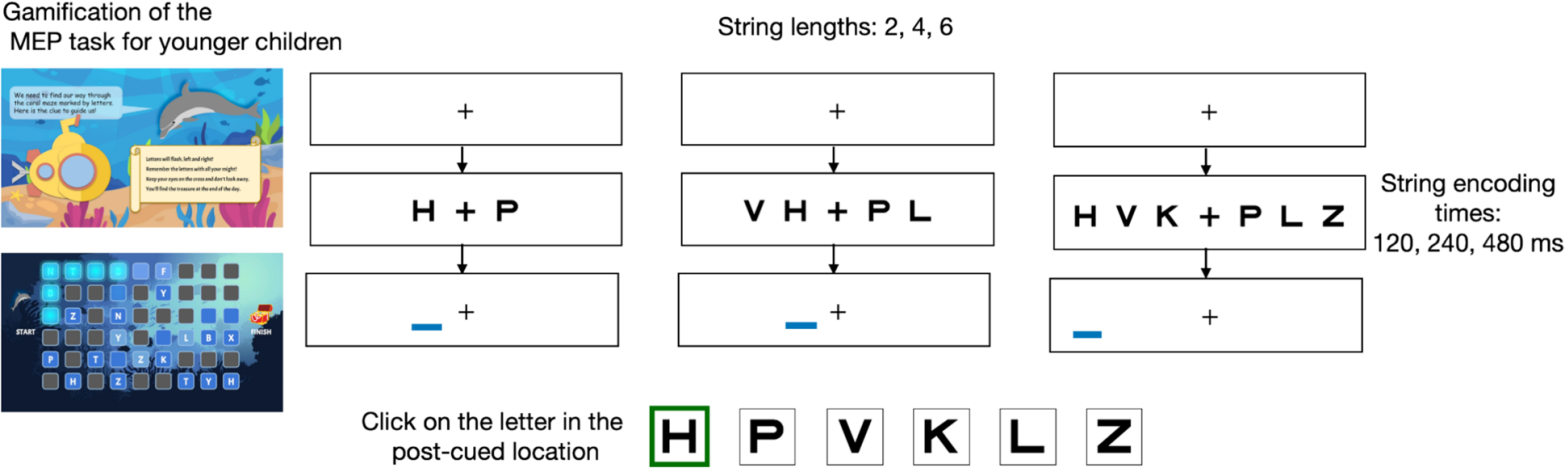
Trial sequence in the partial report version of the MEP task. We modified two dimensions of the MEP task to achieve similar performance levels across different age ranges of the population: the encoding time of the string of letters and the number of elements in the string

**Fig. 2:**
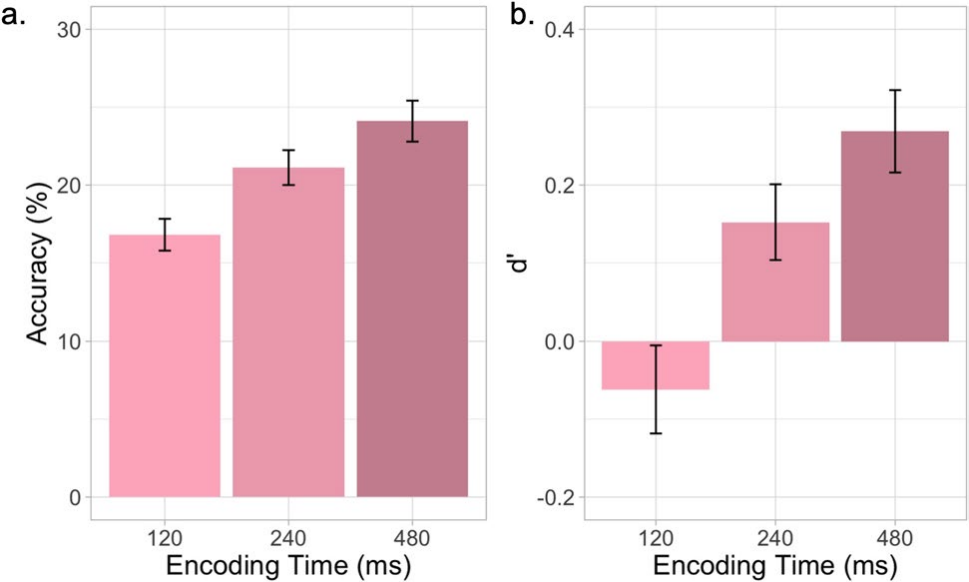
Task performance and encoding time.** a** How K/1 children perform in the MEP task with six-element strings and encoding times of 120, 240, and 480 ms; **b** Performance (*d′*) for each trial condition. Data show that 5–7-year-old children performed at chance, with an encoding time of 120 ms and string length of six letters. Performance increased above chance at longer encoding times, but the task was still very difficult. Error bars represent one standard error of the mean

**Fig. 3:**
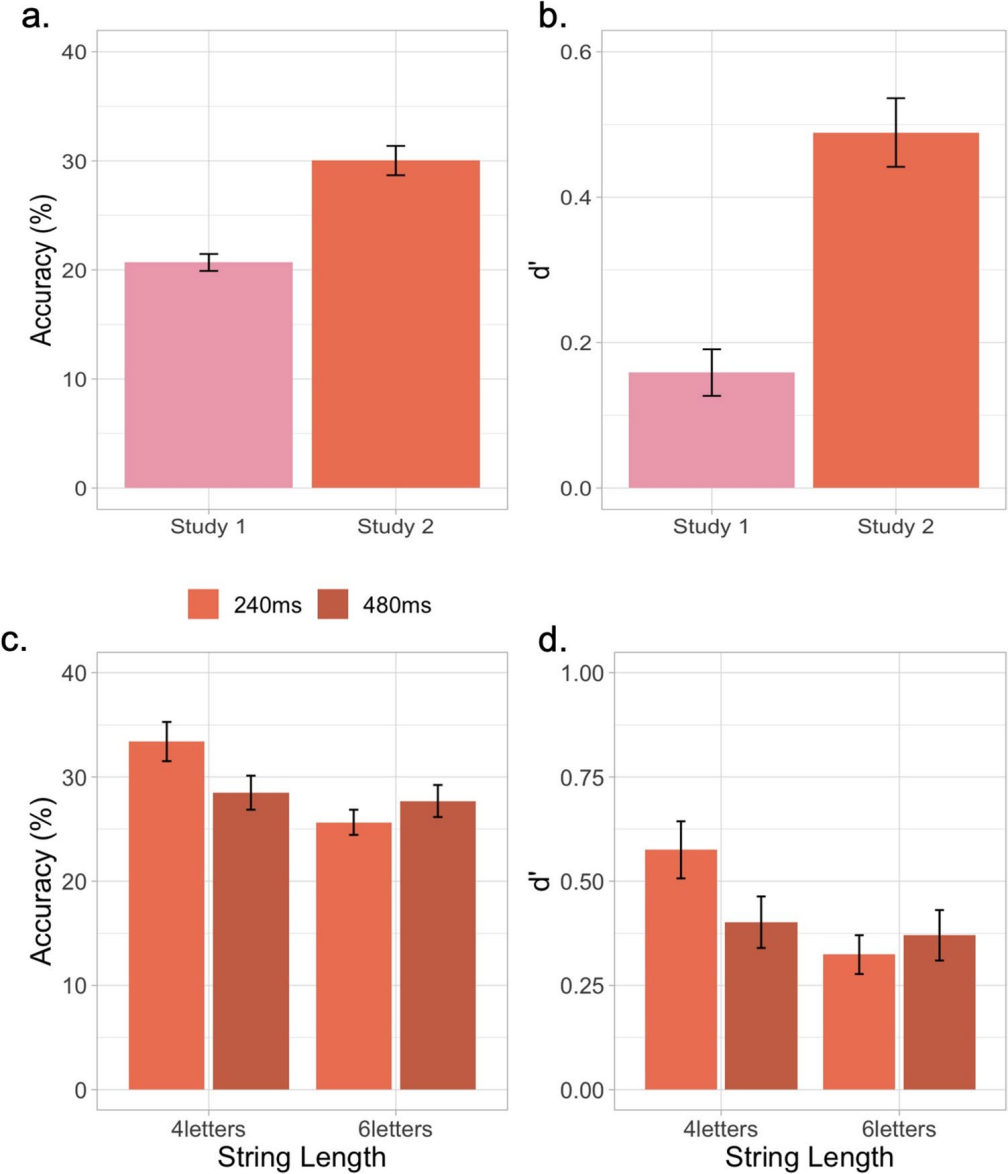
Task performance depends on string length and encoding time. **a**, **b** Overall task accuracy and *d′* from Study 1 and Study 2. Including trials with four elements and cutting down the shortest encoding time of 120 ms seem to have improved performance in the MEP task in Study 2. **c**, **d** Task accuracy and *d′* for each string length and its corresponding encoding time. Note that an encoding time of 480 ms did not improve task performance. Error bars represent one standard error of the mean

**Fig. 4:**
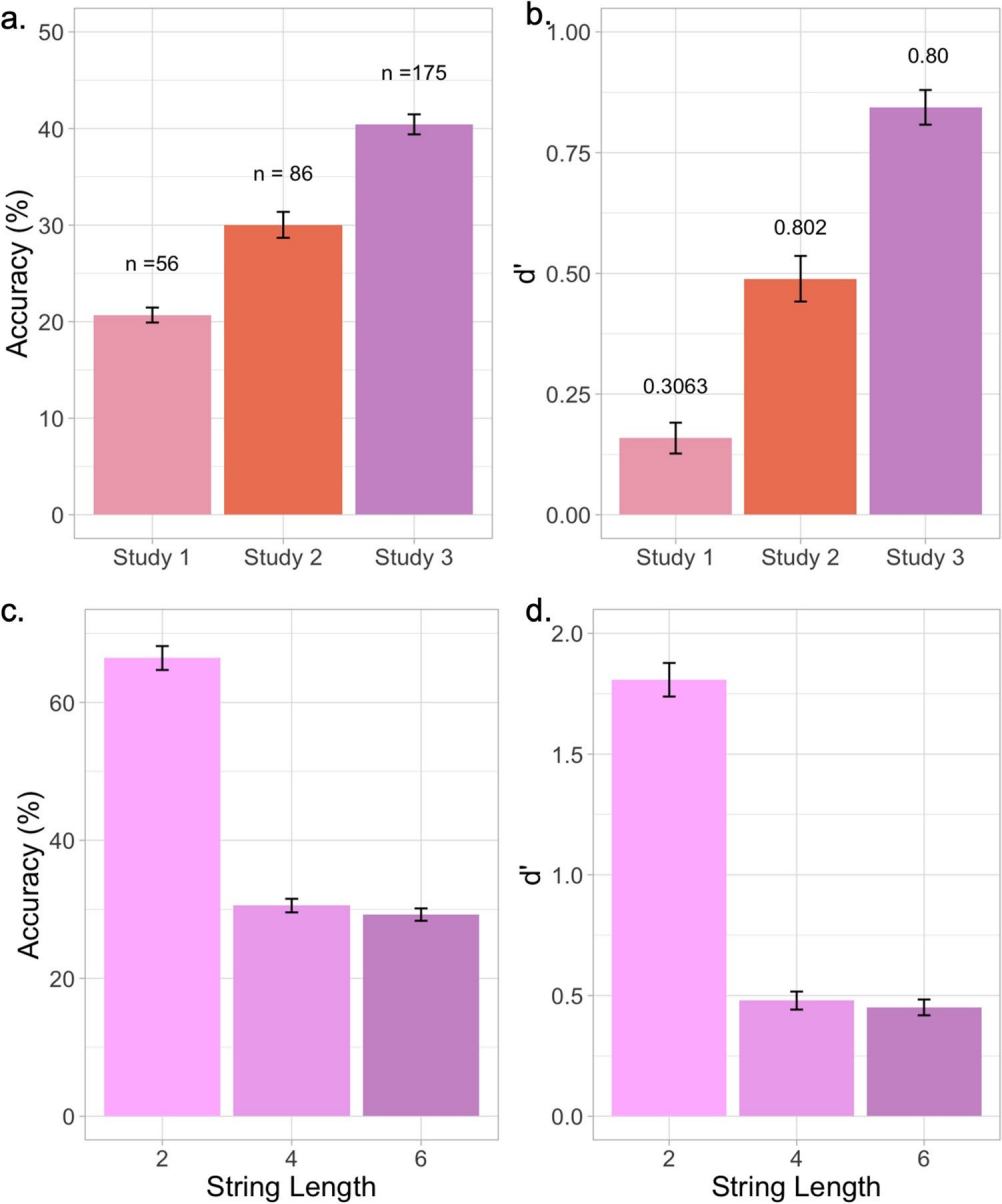
Task performance with three string lengths and one encoding time.** a**, **b** Accuracy and *d′* improvement across different cohorts. Spearman–Brown-corrected split-half reliability for each study is provided in **b**. With the addition of two-letter strings, we observe a significant improvement in performance without compromising task reliability. **c**, **d** Performance across each trial condition in Study 3

**Fig. 5:**
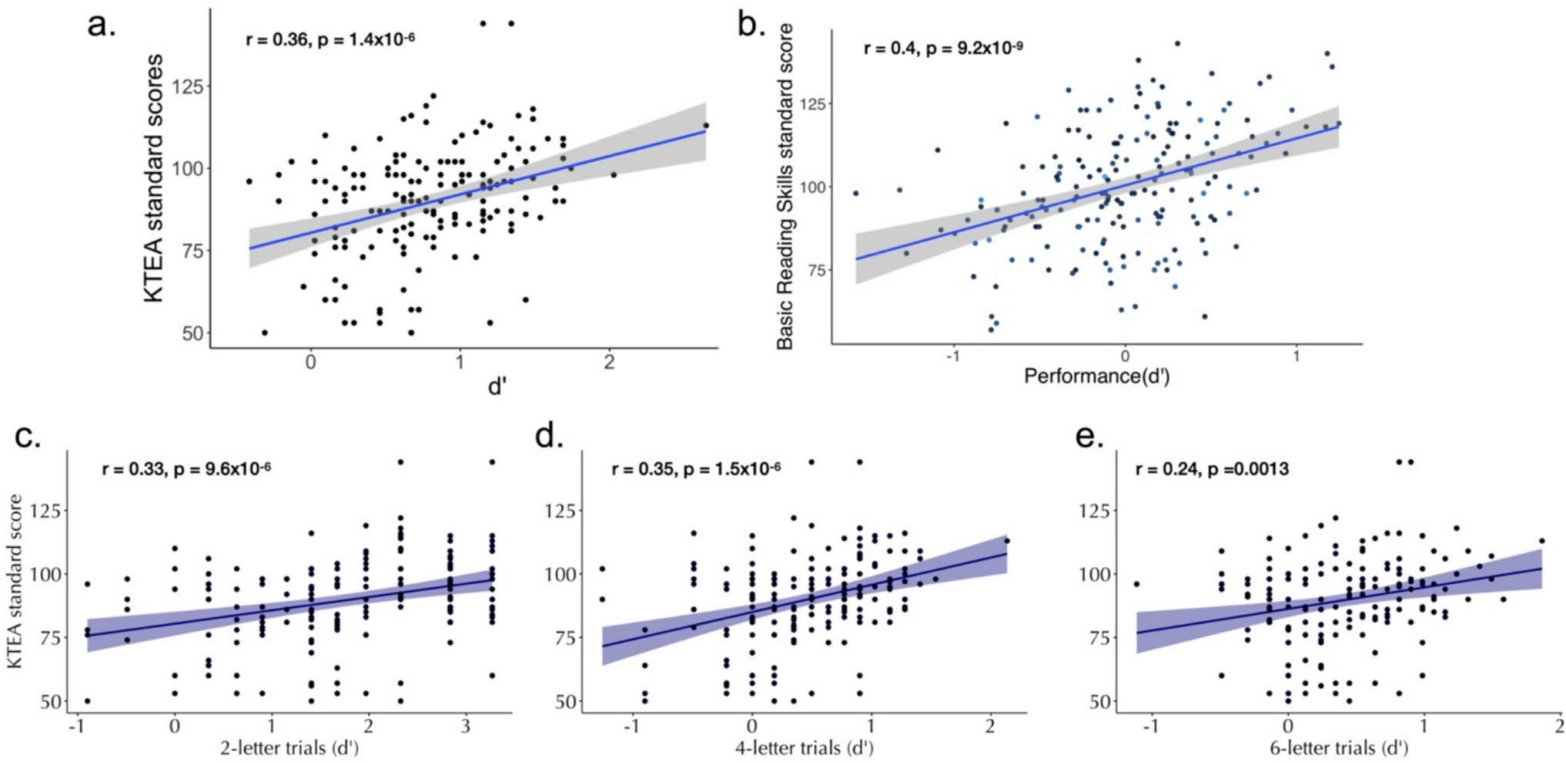
MEP performance predicts reading skill.** a** Correlation between performance in the MEP task and KTEA standard scores. **b** Cross-sectional sample reported in previous work (reproduced from Ramamurthy et al., 2023). **c**–**e** Correlation between task performance in the two-, four-, and six-letter trials and KTEA standard scores

**Fig. 6:**
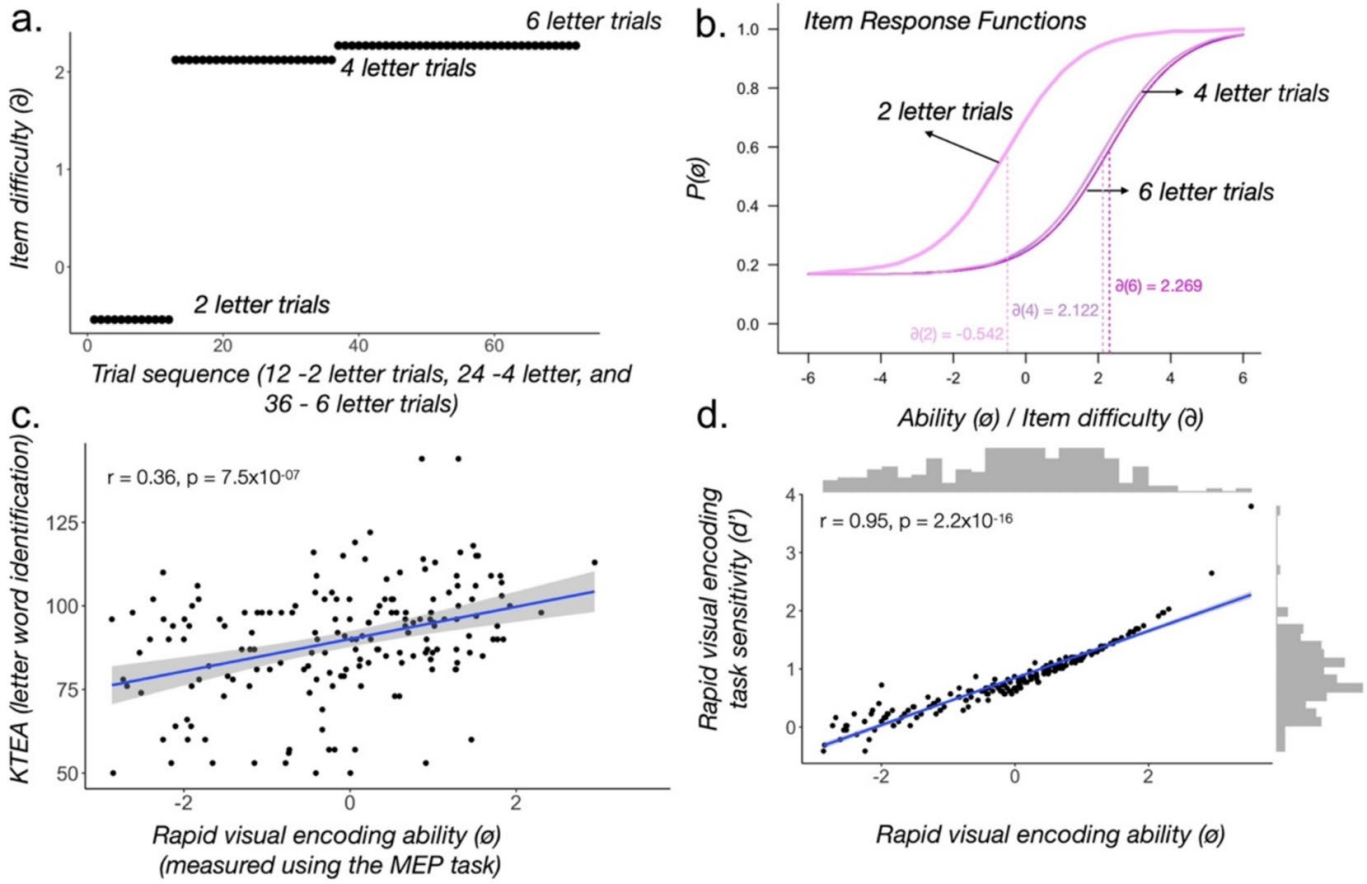
Item response theory (IRT) analysis of MEP data. **a** Item difficulty as a function of trial sequence.**b** Item response functions for all three blocks of different string lengths. **c** Correlation between KTEA and ability estimates. **d** Correlation between performance in the *d′ *space and ability estimates from the IRT analysis

**Fig. 7:**
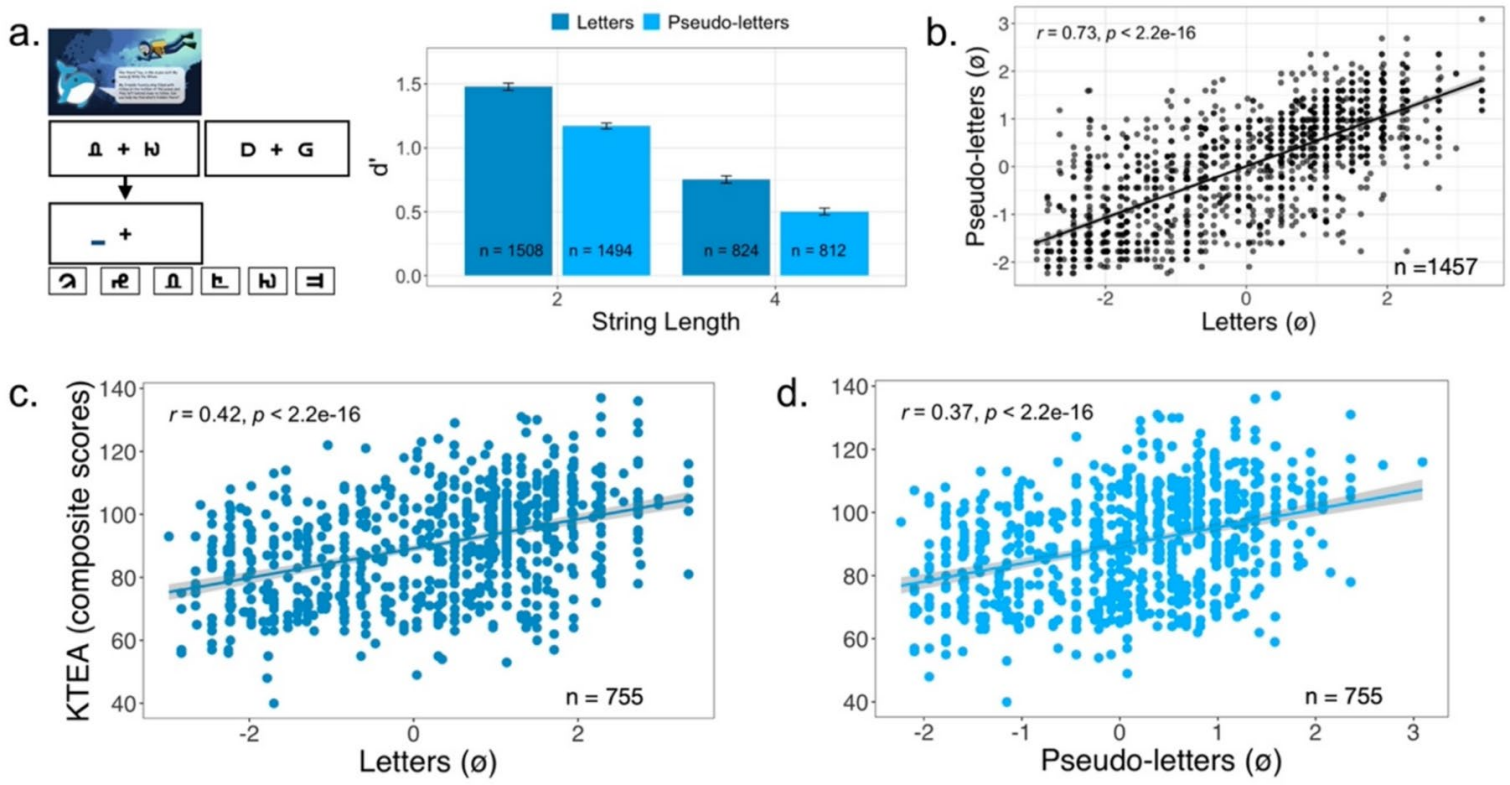
MEP task performance in the K/1 population with letters and pseudo-letters. **a** Trial sequence in the pseudo-letter condition and performance across different string lengths in both versions of the MEP task. **b **Correlation between the letter and the pseudo-letter versions. **c**, **d** Correlation between task performance and reading outcome measured using the composite of three subtests of the KTEA assessment battery (see Methods for details)

In this section we describe the school recruitment process, general data collection, the visual measures and reading outcome measures administered, and general data analysis. In the Methods section below each study, we specify the sample size and task modifications and task administration. Note that Studies 1–4 involved the administration of only the letters version of the MEP task (see below for task description), with the primary goal of modifying the MEP task to best suit the younger population. In Study 5, we administered the optimized version of both the letter and the pseudo-letter version of the MEP task. Under each study, the introduction section provides the aim of each study. The studies progressed systematically: Studies 1–3 focused on iterative task modifications to achieve performance and reliability comparable to older populations, Study 4 employed item response theory analysis to reduce task redundancy and length, and Study 5 assessed the optimized task's relationship with end-of-year reading ability in K/1 children. See supplementary Table 2 for a summary (Figs. 1, 2, 3, 4, 5, 6 and 7).

### Participating school recruitment

Participants were recruited by the University of California, San Francisco (UCSF) Dyslexia Center as part of a California state initiative to develop a universal dyslexia screener. Studies 1–3 differed among the participating school districts. Since the goal was to test how overall task performance changed with task modifications, Study 1 involved only one school, Study 2 involved three schools, and Study 3 involved three schools. Because the goal of the first three studies was to optimize task performance and reliability, we did not collect any other child-specific demographic information.

Study 5 involved 20 schools across the state. The sample for Study 5 was drawn from schools in locations and communities intended to be representative of the state in terms of race, ethnicity, socioeconomic status, and home language (see Supplementary Table 1). Care was taken to reach remote rural, urban, and suburban communities to ensure that the sample was truly representative of the diversity of lived experiences in California. The study aimed to reduce selection bias with a passive consent process. The Institutional Review Board of UCSF determined that a process of informing parents about the study and how their children would be participating, with clear instructions on how to opt out through communications with their school administrators, was appropriate for the research into a universal screener. Parents of eligible kindergarten, first-, and second-grade students received an information sheet describing the project, which involved normal classroom activities and presented no more than minimal risk to participants. Children whose parents wished to opt out did not participate in study activities.

### Measure administration

A team of 3–10 proctors administered these assessments to kindergarten and first-grade students in each of the participating schools based on the school’s convenience. A battery of language measures and reading outcome measures were individually administered to each student. In each school, kindergarten and first-grade children were brought in batches to complete a battery of language, reading, and visual measures. All children were assigned a unique student tracking ID and were randomly grouped in batches. Thus, missing data means that a child either was absent on the day or did not wish to participate.

***Visual measures*** were designed as online games that do not require a test administrator, but proctors got children set up, monitored their focus, and were available to answer questions or assist with technical difficulties. All visual measures were completed on touchscreen Lenovo 13″ Chromebooks that were placed at arm’s distance from children (~ 45–50 cm), and all instructions were narrated by engaging characters that gamified the task for children. Since Studies 1–3 focused on task optimization, two dedicated proctors brought children in batches of four students at a time to complete the task in a separate quiet room. In Study 5, the optimized version was administered to a maximum of 16 children in a dedicated classroom with at least three proctors per grade.

### Task description

 *Rapid visual processing of letters:* The experiment was built using jsPsych (de Leeuw et al., 2023), hosted on Google cloud, and data were written to Google Firebase. The assessment was gamified to make it more engaging and fun for children. The game sets children on an under-the-sea mission in a submarine to help a lost dolphin. The submarine navigates by solving the letter game. In the letter game, children fixate on the center of the submarine porthole, and while they fixate, a string of two letters will appear, one on either side of the fixation, or four letters with two on either side of the fixation, for a brief period (240 ms). After the letters disappear, a post-cue appears at one of the letter positions, and the children’s task was to report the letter that was presented at that location from a choice of six letters that children could tap on. The target strings never formed a word and were randomly sampled without replacement from a set of six consonant letters (K, D, P, F, G, H) that match on perimetric properties (Castet et al., 2017),. All letters were in Sloan font at 100% contrast. Letter height was set to 0.5°, and the center-to-center letter spacing was 0.58° as reported in Ramamurthy et al. (2023). Studies 1–3 administered the letter version of the task.

*Rapid visual processing of pseudo-letters*: This task was identical to the letters described above, except that letters were replaced with pseudo-letters (unnamable abstract symbols) using the Pseudo Sloan font developed to match the letters in terms of perimetric complexity (Vildavski et al., 2022). While single-letter tasks are relatively low in linguistic load, alphabetic characters can still carry learned associations tied to print exposure and orthographic familiarity. By using pseudo-letters that preserve visual complexity while removing familiar symbolic content, we aim to reduce these influences and better isolate domain-general visual processing. This game sets children in an under-the-deep-sea mission where they are scuba divers on a mission to help a lost whale. Given the game’s deep-sea settings, children are instructed that the letters no longer look like letters and are instead patterns (e.g.,  ). The task is identical to the letter task in all other aspects and is always administered after the letter game. The pseudo-letter version was administered only in Study 5.

*Trial sequence:* Figs. 1 and 7a shows an illustration of the trial sequence in the MEP tasks. On each trial, a string of two (or four or six) elements appears for a brief 240 ms. Immediately after the elements disappear, a post-cue (a blue line) appears randomly under one of the element positions. The participant’s task is to report the identity of the target element that was at the post-cue location, by clicking on one letter from a set of six letter choices provided.

### Reading outcome measures

Kaufman Test of Educational Achievement (KTEA) standardized reading assessments consisting of phonological processing, letter naming, and word reading was administered to all children at the end of the school year. These measures are combined to form the reading composite for grades K, 1, and 2. These subsets are designed to measure letter knowledge, phonics/letter–sound correspondence, word reading, and rapid automatic naming skills (Breaux, 2021).

### Data analysis

We used the Palamedes function PAL_SDT_MAFC_PCtoDP (Prins & Kingdom, 2018), which converts proportion correct into *d′* (a measure that quantifies how well an individual is able to discriminate task relevant information from random, irrelevant information). A *d′* of zero means that the ability to discriminate signal from noise is no better than chance, and a higher *d′* means higher discrimination ability, for a standard *M*-alternative forced-choice task, assuming an unbiased observer. For accuracy at floor (0%), we assume that had we run twice as many trials, one trial would be correct, so accuracy = 1/(2 × number of trials). When at ceiling (100%), we assume that had we run twice as many trials, there would be one incorrect trial, so accuracy = 1 – (1/[2 × number of trials]).

We used linear mixed-effects (LME) analyses and post hoc *t*-tests to analyze accuracy across different trial conditions. The random effects consist of subject-dependent random intercepts and slopes. We used a maximal random effects structure (random intercepts and slopes; Barr et al., 2013) in all our LME models unless specified.

### Reliability

For each participant, for each encoding time and string length condition, we computed *d′* separately on odd and even trials. The correlation across subjects between the split-half *d′* levels indicate reliability. A Spearman–Brown correction was applied to the obtained correlation to adjust for the fact that only half the trials were used. For IRT models, we report empirical reliability that is comparable to the split-half reliability estimates. All reliabilities are added to Supplementary Table S2.

### Data-informed design changes to develop a reliable task for kindergarten and first-grade children

#### Study 1: MEP task performance and encoding time

Study 1 set out to determine if the MEP task was feasible in grades K/1 and to explore whether adjustments to target presentation duration could improve performance, leading to a more reliable measure.

#### Study 1: Methods

We first administered the letters version of the MEP task with similar parameters as were used in our previous study of older children and adults (Ramamurthy et al., 2023) (encoding time of 120 ms and string length of six letters). The task was gamified to be engaging for grades K/1 with reward animations as a participant progressed through the task (see Supplementary Material for descriptions of the game). The task was administered to K/1 children (~ 5–7-year-olds, *N* = 56) on chromebooks in a school setting by trained proctors. The number of response choices were reduced to six alternative forced choices (compared to the 12-alternative forced choices employed in the previous study) to reduce the cognitive demands for the younger participants. The total number of trials was based on the three encoding times, six letter positions, and four repetitions for each position, totaling (3 × 6 × 4 =) 72 trials. Studies of short-term visual working memory and visual temporal processing have been consistently reported to be lower/slower in young children (Avant & Thieman, 1985; Avant et al., 1977; Hitch et al., 1988), so we added two longer encoding times of 240 ms and 480 ms in addition to 120 ms and ran 56 participants in a school setting.

#### Study 1: Results

We observed that K/1 children’s task performance was significantly lower with an encoding time of 120 ms (16.813% ± 7.654; *d′* = − 0.062) with very low reliability (Spearman–Brown-corrected split-half reliability = 0.075) compared to the older cross-sectional population reported in our previous work (37.148% ± 1.191; reliability = 0.8). However, performance increased with an encoding time of 240 (21.125% ± 8.371; *d′* = 0.152) and 480 ms (24.107% ± 9.851; *d′* = 0.269). Notably, even at 480 ms, many participants still performed at chance level, and reliability was still low (Spearman–Brown-corrected split-half reliability was 0.306 for the full Study 1 task, with reliability of close to 0 for 120 ms, 0.069 for 240 ms, and 0.309 for 480 ms).

#### Study 2: Do changes to encoding time and string length improve performance in young children?

Encoding a string of six elements simultaneously in a brief time is a challenging task, and Study 1 demonstrated that this task is too hard for young children, even with a longer encoding time. In the next iteration, we modified string length in addition to the encoding time. The primary goal was to improve overall task performance by increasing encoding time and/or decreasing string length.

#### Study 2: Methods

We tested how performance changes in trials with four elements (two on either side of fixation) and six elements (three on either side of fixation) with encoding times of 240 and 480 ms. Trials with four- and six-letter strings were blocked, and within each block the encoding times were randomized. Thus, we had two durations, two string lengths, and four repetitions per target position within the string; therefore, the four-letter block consisted of 2 × 4 × 4 = 32 trials, and the six-letter block consisted of 2 × 6 × 4 = 48 trials, totaling 80 trials. We had a cohort of 86 K/1 children for Study 2 across two different school districts.

#### Study 2: Results

Four-letter strings are cognitively less demanding than six-letter strings. Starting the experiment with easier trials also helps children get familiar with the task before the harder trials start; the outcome of adding four-letter trials to the experiment is reflected as overall higher performance in Study 2. Figure 3a and b show an overall improvement in task performance in Study 2 (mean accuracy = 30.025 ± 1.344; mean *d′* = 0.489 ± 0.047) compared to the overall task performance from Study 1 (mean accuracy = 20.685 ± 0.777; mean *d′* = 0.159 ± 0.032). Figure 3c and d show task performance in the four- and six-element trials with an encoding time of 240 and 480 ms, respectively. An LME fit to performance (*d′*) data with string length and encoding times as fixed effects and subjects as random effects shows a significant main effect of string length [*F*(1, 340) = 18.693; *p* = 2.020 × 10^−5^] and encoding time [*F*(1, 340) = 7.380; *p* = 0.0069] and a significant interaction between encoding time and string length [*F*(1, 340) = 7.765; *p* = 0.006], suggesting that with a string length of four elements, performance is higher with an encoding time of 240 ms compared to 480 ms; however, no such difference in performance between 240 and 480 ms was observed with a string length of six elements (see Fig. 3c, d). Overall task reliability in Study 2 was 0.802; the Spearman–Brown-corrected split-half reliability for the four-letter trials with an encoding time of 240 ms was 0.314 and − 0.176 for the 480-ms trials. The split-half reliability of the six-letter trials with an encoding time of 240 ms was 0.575 and 0.083 for an encoding time of 480 ms. Item-specific reliabilities are not particularly informative in our case, as trial counts vary across string lengths—a deliberate design choice to ensure at least four repetitions per target position—making item-wise reliabilities not very informative; therefore, we report overall reliability for each study.

#### Study 3: MEP task performance with one encoding time and three string lengths

As our primary goal was to be able to optimize for overall task performance and reliability by either increasing encoding time or decreasing string length, we kept an encoding time of 240 ms, as 480 ms did not improve task performance further (as reported in Study 2). We further added a string length of two elements to make the task even easier. Thus, in Study 3, we set out with one encoding time and three string lengths.

#### Study 3: Methods

In the next iteration (*N* = 175, kindergarten and G1 children), we added a two-letter string in addition to four- and six-letter strings. We further reduced redundancy by removing an encoding time of 480 ms that did not increase accuracy. An encoding period 240 ms ensures that encoding occurs without making a saccadic eye movement (Li et al., 2021). Thus, we had one duration, three string lengths, and six repetitions per target position within the string; therefore, the two-letter block consisted of 2 × 6 = 12 trials, the four-letter block consisted of 4 × 6 = 24 trials, and the six-letter block consisted of 6 × 6 = 36 trials, totaling 72 trials.

#### Study 3: Results

Figure 4a and b show that overall task performance increases significantly compared to Studies 1 and 2. Performance in Study 3 (40.429% ± 1.0368; *d′* = 0.844 ± 0.0359; split-half reliability = 0.82) was significantly higher than in Study 2 (30.0252 ± 1.344; *t*(191.2) = 6.128, *p* = 4.974 × 10^−9^). The overall task performance in Study 3 was comparable to a previous study with cross-sectional data (*n* = 185) (Ramamurthy et al., 2023), where overall task performance for 6- to 17-year-olds in the MEP task with an encoding time of 120 ms and a string length of six elements was reported to be 37.148% ± 1.191 (*d′* = 1.160 ± 0.040). Figure 4c and d show the breakdown of performance across different string lengths. Overall task reliability was comparable between Study 3 (*r* = 0.8) and Study 2 (*r* = 0.802) (see Fig. 4b). The Spearman–Brown-corrected reliabilities for each block were 0.473 (two-letter block), 0.657 (four-letter block), and 0.652 (six-letter block). It should be noted that the correlations between the two-, four-, and six-letter blocks were all comparable (between the two-letter and four-letter blocks, *r* = 0.623, *p* < 2.2 × 10^−16^; between the two-letter and six-letter blocks, *r* = 0. 593, *p* < 2.2 × 10^−16^; and between the four-letter and six-letter blocks, *r* = 0.577; *p* < 2.2 × 10^−16^), suggesting that despite huge performance differences across different string lengths, performance was similarly correlated across these blocks.

All children in Study 3 were also administered a subset of the Kaufman Test of Educational Achievement (KTEA) standardized reading assessments that consisted of a letter and word identification task and a pseudo-word decoding task. Of this, only the letter word identification task was completely available across both grades, so we used this measure as a reading outcome measure to investigate the correlation between MEP task performance and reading ability tests that were concurrently administered. In Fig. 5a, we show the relationship between the MEP task performance and KTEA letter word identification scores (grade-standardized). The correlation between task performance in the MEP task and reading outcome measures was *r* = 0.36 (*n* = 175, *p* = 1.4 × 10^−6^), which is comparable to the correlation observed in our previous study of older children aged 6–17 years (*r* = 0.40) (Ramamurthy et al., 2023). When we separately examine the performance of two-, four-, and six-letter trials in relation to KTEA, we find similar effects for each string length (though six-letter trials have a slightly lower correlation than two-letter and four-letter trials; see Fig. 5c–e).

Results from Study 3 suggest that we have arrived at a task that is well suited for younger children and is comparable to previous data from older children in terms of (a) the magnitude of overall task performance and reliability and (b) the strength of correlation with standardized measures of single-word reading (Fig. 5). However, the total task duration was ~ 12 min, which is too long for this age range, particularly for use in a school setting. To further reduce redundancy and to increase the test discriminability across a wide range of ability levels, we turned to item response theory (IRT) to optimize the task further.

#### Study 4: Item response theory (IRT) analysis to optimize trial structure

Adaptive methods in psychophysics (Green et al., 1989; Leek, 2001; Lesmes et al., 2010; Rinderknecht et al., 2018; Solomon, 2011; Watson & Pelli, 1983) have largely evolved for the purpose of increasing efficiency by optimizing experiment time while not compromising accuracy or reliability. One of the key assumptions to be able to effectively apply adaptive psychophysics methods is to ensure a monotonic relationship between stimulus strength and performance. In our series of studies, we monitored performance changes in relation to encoding time and string length in the MEP task. From Study 3, we can conclude that increasing the string length increases task difficulty. However, the number of stimulus levels along this dimension is only three: two-, four-, and six-letter strings. Using any of the conventional adaptive psychophysical methods to make the task more efficient for the younger children would mean that stimulus presentation would start with one of these string lengths, and on each trial the number of elements in the string would vary to converge on the most informative trials. For a task like the MEP task and a population cohort as young as K/1, the adaptive procedure could be cognitively demanding, as trials would randomly vary in string length and therefore the trial-to-trial difficulty. To avoid further task complexity, we used an IRT analysis to calculate the difficulty of two-, four-, and six-letter strings and then designed some simple adaptive rules to optimize the total duration of the task.

The core idea of IRT is to describe how participant attributes (e.g., rapid visual processing ability) and item characteristics (e.g., string length) contribute to the probability of a correct response. As a participant's ability increases, the probability of a correct response also increases (see Carlson, 2020; Chalmers, 2012; Rasch, 1993). Likewise, as the difficulty of an item increases, the probability of a correct response decreases.

#### Study 4: Methods

Data from Study 3 (*N* = 175) were used to calibrate an IRT model. Trials with different string lengths were blocked (12 two-letter trials, 24 four-letter trials, and 36 six-letter trials). The goal of IRT is to place item difficulty (blocks of different string lengths) on an interval scale. The Rasch model (one-parameter logistic with a guess rate fixed at 0.167) was fit to the response data for the three item types (constraining difficulty for repeated trials with the same string lengths) for all 175 participants using the mirt package in R (Chalmers, 2012). The Rasch model uses a logit scale, which is a logarithmic transformation of the odds of success in a binary response on each trial with a certain item difficulty level. The ability estimates are on logit units and represent the location of individuals on the latent trait continuum.

#### Study 4: Results

Figure 6a shows the item difficulty for the three item types (two-, four-, and six-element lengths), and Fig. 6b shows the item response function of each block (each string length). Consistent with performance data (*d′*, see Fig. 4d), item response functions also show that two-element trials are the easiest, and the four- and six-element trials have similar item characteristics. Therefore, as a first step towards reducing redundancy, we can shorten the task by eliminating the 36 six-element trials. In addition, the fit of individual items to the Rasch model was assessed using the chi-square-based item fit statistics. Among the 72 items, the root mean square error of approximation (RMSEA) values ranged from 0.000 to 0.145, with a median of 0.051, suggesting that most items demonstrated adequate fit; *p* values ranged from < 0.001 to 0.847, with a median of 0.030. However, approximately 12 items showed evidence of poor fit, characterized by elevated RMSEA and low *p* values. These items were removed in the optimized version of the model to ensure alignment with the Rasch model’s assumptions. Importantly, the empirical reliability as measured by the IRT model is 0.86 for the K/1 population, which is comparable to the reliability reported in the cross-sectional data between 6 and 17 years reported in the previous study. Notably, the correlation between KTEA (letter word naming ability) and ability estimates in the MEP task showed the same correlation strength (*r* = 0.36, *p* = 7.5 × 10^−7^) (see Fig. 6c). Ability estimates and average *d′* values were highly correlated (*r* = 0.95, *p* = 2.2 × 10^−16^), as shown in Fig. 6d.

#### Study 5: IRT-optimized MEP task as a K/1 screener

Study 5 capitalized on the insights from Studies 1–3 and the IRT model from Study 4 to develop an optimized version of the MEP task, quantify reliability in a large and diverse sample of K/1/2 students, and examine the predictive validity for end-of-year reading scores. Study 5 aims to empirically compare the strength of the correlations between task performance and end-of-year reading scores in the optimized versions to that reported in the previous study on cross-sectional data from 7- to 17-year-old participants (Ramamurthy et al., 2023).

We used the optimized version of the MEP task described below and created two versions of the MEP task. Both versions were identical in trial structure and trial parameters, except that the elements were letters in one and pseudo-letters in the other (PseudoSloan that matched with letters in their ink area). The primary motivation for using pseudo-letters, which are unknown visual patterns, is to develop a language-agnostic measure that can potentially be used across cultures. In 3 months (in winter 2023), the letter and pseudo-letter versions of the MEP tasks were administered to ~ 1,550 children in K/1 across the state of California by the UCSF Dyslexia Center as part of an initiative to develop a universal dyslexia screener. This project allowed for data collection in a large and diverse K/1/2 sample across the state of California. We aimed to address two questions in this administration: (1) How do the optimized MEP tasks—letter and pseudo-letter versions—compare with each other? (2) How does performance in both versions of the task predict end-of-year reading outcomes (KTEA scores)?

#### Study 5: Methods

IRT analysis showed that item difficulties of the four- and six-element strings were similar. To reduce redundancy, we eliminated the six-element trials completely and used two- and four-element trials with an encoding time of 240 ms. Examining the theta distribution in Study 4 revealed that two-element trials were more informative than four-element trials for most K/1 participants. Thus, we start the experiment with 24 two-element trials. The choice of 24 trials in the two-element (easy) block is driven by a simulation of three different respondents with performance patterns (HP = high performance, LP = low performance, MP = mixed performance), creating synthetic response patterns where HP participants consistently answer correctly, LP participants consistently answer incorrectly, and MP participants show a mixed pattern. We observed that the theta estimates stabilize around 16 trials (see Supplementary Fig. 2). We kept the trial length to 24 trials, as children need a few practice trials to master the task. In addition, for efficient task administration, we created a simple transition rule and a termination rule. At the end of the two-element block, if children had four or more trials correct (≥ 4/24), then they transitioned to the next item difficulty and completed eight four-letter trials. If children performed at or below chance, then the task terminated after 24 two-element trials. If they performed above chance, they proceeded to the more difficult four-element block. We chose eight trials for this block, keeping in mind the total duration of the task. Together, the total test duration was significantly reduced to a median time of 5.698 min ± 0.1 for the letter version and 5.291 min ± 0.058 for the pseudo-letter version (see Supplementary Figure S1). A total of 1,606 children attempted the MEP tasks, and 98 children did not reach the task end screen for various reasons. The KTEA distribution of the children who were excluded from further analysis is presented in Supplementary Figure S3. We observed that those excluded did not show any unique reading profiles.

#### Study 5: Results

Figure 7a shows a trial sequence in the pseudo-letter version of the task and summarizes task performance in the letter and pseudo-letter versions. An LME fit to performance (*d′*) data with string length (two and four) and stimulus type (letters, pseudo-letters) as fixed factors shows a significant main effect of string length [*F*(1, 3,767) = 248.3; *p* = 3.175 × 10^−54^] and stimulus type [*F*(1, 3,767) = 55.689; *p* = 1.048 × 10^−13^] with no significant interaction [*F*(1, 3,767) = 2.525; *p* = 0.112]. Overall, performance (*d′*) in the letter task was higher than the pseudo-letters [*t*(3,767) = 7.463, *p* = 1.048 × 10^−13^], and performance in the two-element condition was higher than in the four-element trials [*t*(3,767) = 15.757; *p* = 1.084 × 10^−54^]. IRT-based theta estimates combine responses across different item difficulties into a single estimate of participant ability. Figure 7b shows theta estimates for 1,457 participants who completed both the letter and pseudo-letter version of the MEP task. Although a universally recognized gold standard for the MEP task does not exist, it is common in such cases to establish validity by comparing new measures to each other, especially when they are designed to assess the same underlying construct. In this study, we found a highly significant correlation between the letter and pseudo-letter versions of the MEP task (*r* = 0.73, *p* < 2.2 × 10^−16^; CI = 0.710–0.758; dis-attenuated correlation *r* = 0.915, with task reliability of 0.80). This strong correlation suggests that both tasks reliably measure the same construct. Of the 1,457 participants, 755 were administered a KTEA composite measure (see Methods section for how composites were computed). The correlation strength between KTEA and task performance in both the letter (*r* = 0.42, *p* < 2.2 × 10^−16^; CI = 0.359–0.477; see Fig. 7c) and pseudo-letter (*r* = 0.37, *p* < 2.2 × 10^−16^; CI = 0.307–0.431; see Fig. 7d) versions were similar [∆*r* = 0.0489; z = 1.9166, *p* = 0.0553; computed using the cocor package in R (Diedenhofen & Musch, 2015) that uses Hittner, May, and Silver's (2003) modification of Dunn and Clark’s *z* (1969) using a back-transformed average Fisher’s (1921) *Z* procedure]. The correlation strengths were also comparable to our Pilot 3 (calibration data set) and the previous cross-sectional study (Ramamurthy et al., 2023).

It is important to note that reading is a complex skill that draws upon multiple cognitive processes, including phonological awareness, vocabulary, working memory, rapid naming, and visual processing. Visual processing is one dimension of a multi-factorial model of reading. The moderate correlation between our measure and KETA scores should be viewed in the context of reading as a combined influence of a myriad of risk factors, where even phonological awareness, often considered the strongest predictor of early reading, typically explains 15–25% of variance in reading outcomes (Hogan et al., 2005). Research supports the notion that no single cognitive predictor typically accounts for more than 10–20% of reading variance (Wu et al., 2020).

A regression model with both letter and pseudo-letter theta estimates predicted KTEA better than either measure on its own: Model A performance in the letter version the MEP task explained 17.5% of the variance in KTEA composite scores; Model B performance in the pseudo-letter version of the MEP task explained 13.7% of the variance in KTEA composite scores; and Model C with both letters and pseudo-letters (KTEA ~ letters + pseudo-letters) as predictors explained 18.6% of the variance in KTEA composite scores. Model C is significantly better than either of the other two models [Model A vs. C: *F*(1, 752) = 10.609, *p* = 0.001; Model B vs. C: *F*(1, 752) = 46.373, *p* = 2.002 × 10^−11^]. Thus, by using IRT to optimize the number of trials and by effectively combining two-element and four-element trials into a single ability estimate, we were able to create a short (~ 5-min), child-friendly measure of rapid visual processing that predicts end-of-year reading scores in kindergarten and first-grade students.

#### Study 5: Discussion

Our approach adapts established developmental assessment principles, modifying tasks to be age-appropriate while maintaining core processing demands. This parallels the evolution of other developmental measures, such as visual acuity tests. We recognize the challenges in establishing construct equivalence across different age groups. The current evidence for task similarity is indirect, as we have not yet conducted formal tests of measurement invariance across different age groups. Future research should administer the same version of the task across the full age range (5–18 years) to enable multi-group confirmatory factor analyses and examination of differential item functioning through item response theory. Such analyses would provide stronger evidence that the modified task measures the same underlying construct across development. Nevertheless, our results set a strong premise to longitudinally investigate if the ability to rapidly process visual elements early in development can predict future reading abilities.

## Conclusion

Growing recognition that early deficits in visual processing might confer risk for dyslexia is reflected in recent dyslexia screening legislation (SB 114: Committee on Budget and Fiscal Review. Education Finance: Education Omnibus Budget Trailer Bill, 2023) that places measures of visual processing alongside conventional screening measures like phonological awareness, rapid automatized naming, and letter–sound knowledge (Ward-Lonergan & Duthie, 2018; Zirkel, 2020). However, to date, very little research has been done to develop and validate relevant measures of visual processing for children as young as kindergarten. It is therefore not sufficient to merely translate visual measures from the literature into screeners, but it is also necessary to investigate if the developed measure can be reliably extended to study relevant visual functions in younger children. In this study, we report a series of informed design changes and validation studies to translate the multi-element processing task (Ramamurthy et al., 2021, 2023; Valdois, 2022) to a younger population in the school setting. We present results from a series of iterative design changes to show how we arrived at an optimized version of the task without compromising on performance and reliability of the measure in the younger age groups. The design changes we implemented for the letter version of the task were extended to the pseudo-letter version that has the benefit of being completely independent of experience with letters or general language experience. Finally, we present results based on administering both the letter and pseudo-letter versions of the MEP task to a large and diverse population of K/1/2 children. We show that even though overall task performance was higher in the letter version compared to the pseudo-letter version, prediction of reading outcomes was similar across both versions of the MEP task. Performance between the letter and pseudo-letter versions was highly correlated, providing evidence that they reliably tap into the same latent construct, making the pseudo-letter version a language-agnostic measure. With quick and reliable measures, we can address long-standing questions about the role of visual processing deficits in reading disabilities. It should be noted that reading disabilities are not primarily a visual disorder; therefore, no visual measure should be conceived of as a standalone screener. Rather, visual processing deficits should be contextualized under the multifactorial model of reading challenges that calls for a comprehensive longitudinal study design to understand how the development of visual deficits, like that presented in this study, intersect with that of the development of reading abilities. Even though the role of rapid visual processing in the broader dyslexia phenotype is far from settled, we provide additional evidence for the utility of visual measures in the context of a universal screener and provide a roadmap for the translation and validation of visual measures to diverse populations of kindergarteners.

## Supplementary Information

Below is the link to the electronic supplementary material.

Supplementary file1 (DOCX 210 KB)

## Data Availability

Data availability is restricted due to legal obligations laid out in data privacy agreements with partnering school districts.

## Code availability

Due to data protection rules, the data cannot be made available, but documented code for the analyses in this article is available at https://osf.io/tj3pc/. The experiment code to replicate the experimental paradigm is available at https://github.com/yeatmanlab/roar-mep.
