# Supplementary material for: Design and validation of a rapid visual processing measure for screening reading difficulties in early childhood
Source: Behav Res Methods. 2025 Jul 25;57(9):237. doi: 10.3758/s13428-025-02739-7 (PMC12296926; doi:10.3758/s13428-025-02739-7)
Supplement: Supplementary file 1 — Supplementary file1 (DOCX 210 KB) [file 13428_2025_2739_MOESM1_ESM.docx]

### Supplementary material.

​​
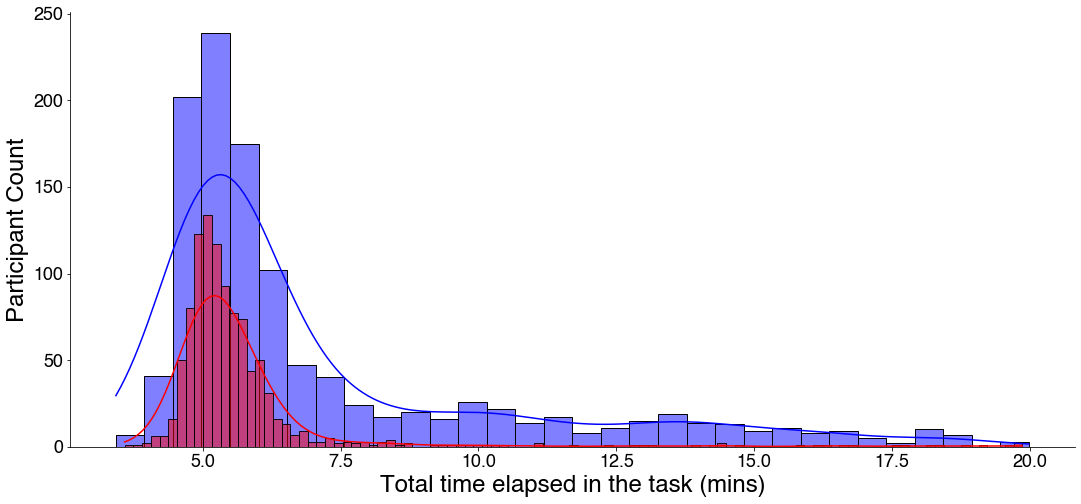


Figure S1. The distribution of the total time taken to complete MEP letters (blue) and pseudo-letters (red) across the entire administration reported in study 5. For some participants the task takes up to 14 mins (more often these were instances when the child was distracted and needed proctors to intervene and get them back to the game. The inference was made based on the proctor record of the time taken at the school administrations).

Figure S2. shows the estimated ability (θ) on the y-axis plotted against trial number on the x-axis, with error bars representing standard errors - HP (red) shows increasing positive estimates, LP (green) shows decreasing negative estimates, and MP (blue) remains closer to the middle. We simulated three different performance patterns (HP: High Performance, LP: Low Performance, and MP: Mixed Performance) across 16 trials, creating synthetic response patterns where HP participants consistently answer correctly, LP participants consistently answer incorrectly, and MP participants show a mixed pattern. For each trial, using mirt package in R, we calculated factor scores (θ) using MAP estimation and their standard errors, tracking how ability estimates and uncertainty change as more responses are observed. The item difficulty of the two-letter trials from Study 4 were used for this simulation. This analysis helps visualize how theta and stand errors stabilize.


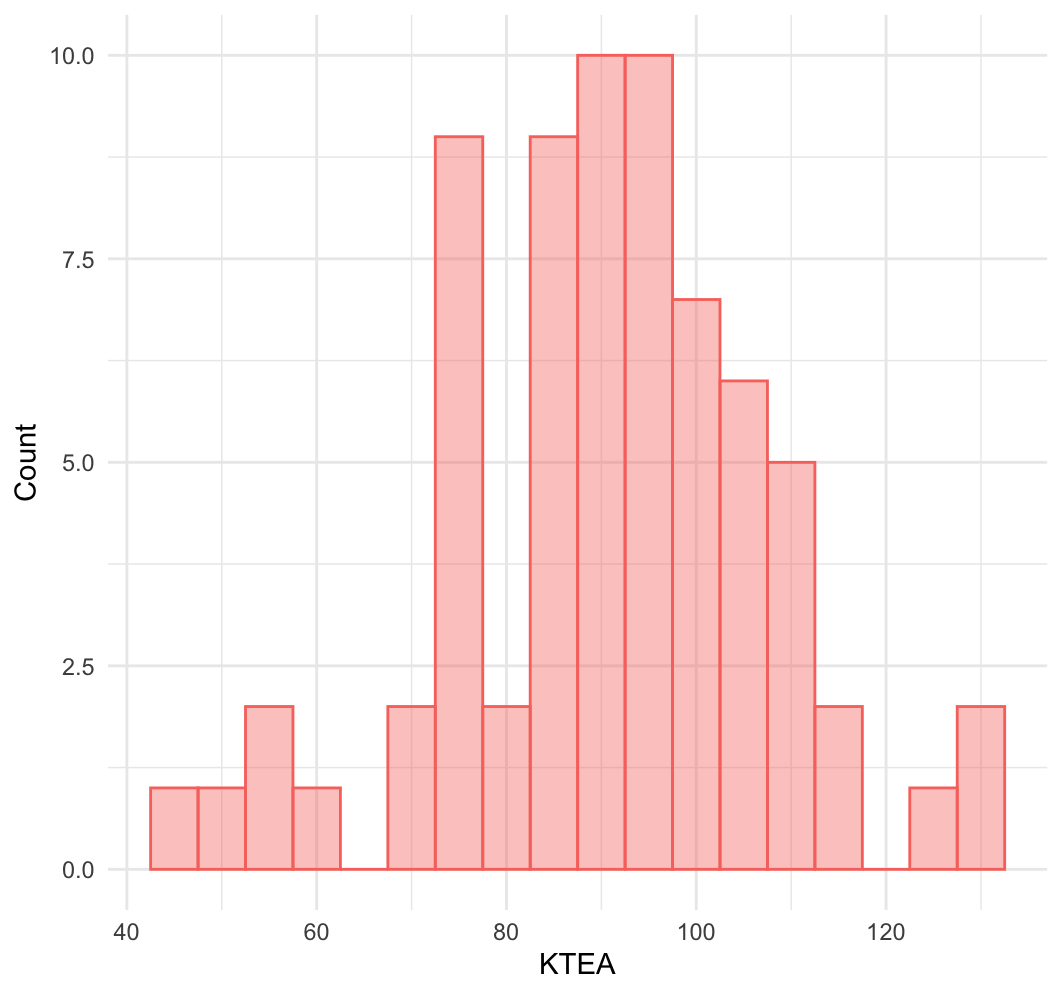


Figure S3. shows the distribution of those children who were excluded from further anlaysis in Study. A total of 98 children did not make to the end screen. This was due to various reasons and a common procotor report was that some children excitedly touched everywhere on the touch screen and swiped themselves out of the game to engage with other things on chromebooks. Interestingly we note no particular bias in the distribution of the KTEA scores from this group.

Supplementary Table 1. Demographic information based on race, ethnicity and gender of the 1466 participants in Study 5 is tabulated below. The percentages can be compared to that reported by the State of California here (<https://www.cde.ca.gov/ds/ad/ceffingertipfacts.asp>).

| **Gender** | **Unique Counts** | **Percentage** |
| --- | --- | --- |
| Male | 647.0 | 44.1 |
| Female | 796.0 | 54.3 |
| Unknown | 23.0 | 1.6 |
| **Ethnicity and race** |  |  |
| Hispanic or Latino | 1007.0 | 68.7 |
| Non-Hispanic Black | 82.0 | 5.6 |
| Non-Hispanic Asian | 53.0 | 3.6 |
| Non-Hispanic Filipino | 38.0 | 2.6 |
| Non-Hispanic White | 207.0 | 14.1 |
| Non-Hispanic American Indian or Alaska Native | 10.0 | 0.7 |
| Non-Hispanic Native Hawaiian or Other Pacific Islander | 4.0 | 0.3 |
| Multiple | 34.0 | 2.3 |
| Unknown | 31.0 | 2.1 |
| **EL status** |  |  |
| English or American Sign Language Only | 719.0 | 49.0 |
| English Learner | 587.0 | 40.0 |
| Initial Fluent English Proficient | 63.0 | 4.3 |
| Reclassified Fluent English Proficient | 38.0 | 2.6 |
| Unknown | 59.0 | 4.0 |

Supplementary Table 2. Summarizes sample size, trial structure, reliability and the reading outcome used to understand the correlation between the visual task performance and reading ability.

|  | Sample Size | Trial structure | Study reliability | Reading outcome |
| --- | --- | --- | --- | --- |
| Study 1 | 56 | (3 encoding times x 6 positions x 4 repeats) = 72 trials | 0.306 | - |
| Study 2 | 86 | 4-letter block: 2 encoding times x 4 positions x 4 repeats  = 32 trials; 6 -letter block: 2 encoding times x 6 positions x 4 repeats = 48; total = 80 | 0.80 | - |
| Study 3 | 175 | 2-letter block: 2 positions x 6 repeats = 12; 4-letter blocks: 4 positions x 6 repeats = 24; 6-letter block: 6 positions x 6 repeats = 36; total= 72 trials | 0.8 | KTEA letter word identification sub test |
| Study 4 | 175 | Uses sample data from study 3 and employees an IRT model | Empirical reliability: 0.86 | KTEA letter word identification sub test |
| Study 5 | 1457 | Optimized task: 24, 2-element trials with a maximum of 8, 4-element trials. Both the letter and pseudo-letters were administered | Dis-attenuated correlation between the letter and pseudo-letter versions = 0.915 | KTEA composite score |
